# Enhanced Potentiometric Hydrogen Sensing Response Based on the Ba_0.5_Sr_0.5_Co_1–*y*_Fe_*y*_O_3–__δ_ Electrode with Unusual Polarity

**DOI:** 10.1021/acsomega.3c06833

**Published:** 2024-02-14

**Authors:** Hong Zhang, Yanqing Liu, Hailin Su, Yuelong Zhu, Haowei Zhu, Shibin Nie, Liangji Xu

**Affiliations:** † Joint National-Local Engineering Research Centre for Safe and Precise Coal Mining, Anhui University of Science and Technology, Huainan, Anhui 232001, P.R. China; ‡ College of Public Safety and Emergency Management, Anhui University of Science and Technology, Hefei, Anhui 231131, P.R. China; § Institute of Energy, Hefei Comprehensive National Science Center, Hefei, Anhui 230051, P.R. China

## Abstract

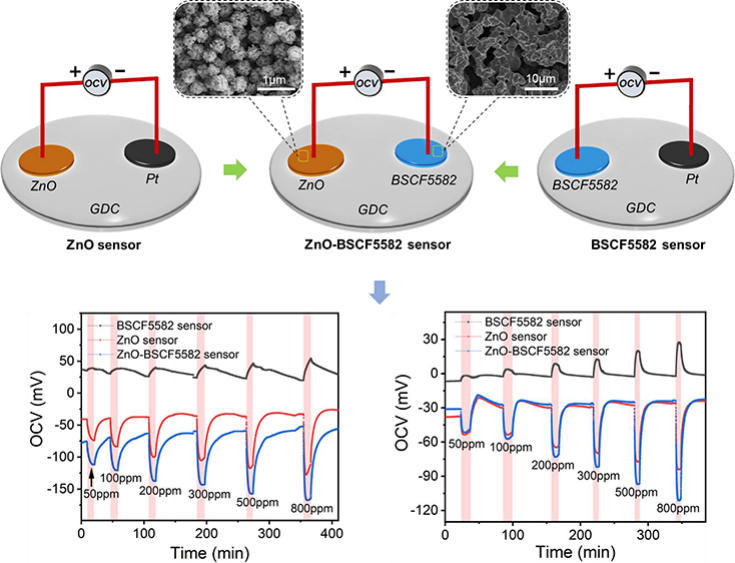

In this work, unusual potentiometric hydrogen sensing of mixed conducting Ba_0.5_Sr_0.5_Co_0.8_Fe_0.2_O_3–__δ_ was reported. Inspired by the unusual polarity, a dual sensing electrode (SE) potentiometric hydrogen sensor was fabricated by pairing Ba_0.5_Sr_0.5_Co_0.8_Fe_0.2_O_3–__δ_ with electronic conducting ZnO to enhance the hydrogen response. Hydrogen sensing measurements suggested that significantly higher response, larger sensitivity, and lower limit of detection (LOD) were achieved by the dual SE sensor when compared with the single SE sensor based on Ba_0.5_Sr_0.5_Co_0.8_Fe_0.2_O_3–__δ_ or ZnO. A high response of 97.3 mV for 500 ppm hydrogen and a low LOD of 2.5 ppm were obtained by the dual SE sensor at 450 °C. Furthermore, the effect of the Fe doping concentration in Ba_0.5_Sr_0.5_Co_1–*y*_Fe_*y*_O_3–__δ_ (*y* = 0.2, 0.5, and 0.8) on hydrogen sensing response was investigated. The potentiometric response values to hydrogen increased monotonically with increasing Fe doping concentration. With the Fe/Co atomic ratio increased from 0.25 to 4, the responses to 500 ppm hydrogen raised by 69.6 and 94% at 350 and 450 °C, respectively. The sensing behaviors of unusual Ba_0.5_Sr_0.5_Co_1–*y*_Fe_*y*_O_3–__δ_ may be ascribed to the predominant surface electrostatic effect. These results show that mixed conducting Ba_0.5_Sr_0.5_Co_1–*y*_Fe_*y*_O_3–__δ_ is desirable for developing high-performance dual SE hydrogen sensors.

## 1 Introduction

Highly sensitive detection of hydrogen is essential to ensure the safety of hydrogen use, production, storage, and transportation.^1−3^ Among many hydrogen techniques, potentiometric hydrogen sensors have attracted tremendous attention due to their simple structure, small size, robustness, and high-temperature resistance.^4,5^ This type of sensor generally consists of a sensing electrode (SE), a reference electrode (RE), and a solid electrolyte. The sensor response is the difference between responses of the SE and RE.^6^ To achieve high sensing response, considerable research efforts have been devoted to SE engineering via the manipulation of SE compositions and morphologies.^7,8^ Less attention has been paid to REs, which is another crucial factor affecting the sensor response. Typically, the RE was made of scarce and expensive platinum (Pt) with little potential change at high temperatures, which makes no significant contribution to sensor response and cost-effectiveness.^9^ Substitution of another semiconductor oxide SE for the Pt RE to fabricate dual SE sensors has been proven to be an effective way to reduce the sensor cost while tuning the sensing performance, such as response, sensitivity, and selectivity.^10−14^ However, conventional dual SE sensors based on semiconductor oxides with the same potential polarity would drastically lower the response and sensitivity, unfavorable for highly sensitive gas detection.

Recently, mixed ionic-electronic conductor (MIEC) SEs with unusual potential responses were used to enhance sensor response by pairing them with a conventional semiconductor oxide SE. For example, Zhang et al. reported hydrogen sensing response enhancement of dual SE sensors based on unusual SrFe_0.5_Ti_0.5_O_3–__δ_ and conventional La_0.8_Sr_0.2_Cr_0.5_Fe_0.5_O_3–__δ_.^15^ Lin et al. found that ammonia sensing response was enhanced by pairing unusual LaCoO_3_ and conventional ZnO.^16^ Nonetheless, so far, there are very limited MIECs known with unusual potential polarities. To realize high-performance hydrogen sensing, it is crucial to develop MIECs with unusual potential hydrogen responses and further construct dual SE sensors.

Ba_*x*_Sr_1–*x*_Co_1–*y*_Fe_*y*_O_3–__δ_ (BSCF), an MIEC perovskite with a high rate of oxygen diffusion, has been widely studied for fuel cells,^17^ gas separation membranes,^18^ and electrocatalysis.^19^ The unusual potential polarity of Ba_0.5_Sr_0.5_Co_0.8_Fe_0.2_O_3–__δ_ (BSCF5582), one of the most typical types of BSCF, to 2-ethylhexanol, ethanol, and other gases, was found previously.^20^ Such unique properties of BSCF5582 may be desirable for enhancing potentiometric hydrogen sensing. Nevertheless, the potentiometric hydrogen sensing performance of BSCF5582 is not clear yet. Moreover, the electronic conductivity, surface chemical state, and thermal expansion coefficient can be greatly affected by varying the Fe doping concentration in the B-site,^21,22^ which may provide an opportunity to optimize the hydrogen response. However, the effect of the Fe doping concentration on the potentiometric hydrogen sensing of BSCF has not been reported.

In this work, the potentiometric hydrogen sensing properties of BSCF5582 were studied. A planar dual SE sensor was fabricated subsequently by pairing BSCF5582 and the usual electronic conducting ZnO on a Ce_0.8_Gd_0.2_O_1.9_ (GDC) electrolyte disk, and the hydrogen response of the dual SE sensor was systematically examined. In addition, the influence of the Fe/Co ratio in BSCF on the sensor response was investigated. The sensing behaviors of BSCF were discussed in terms of the predominant surface electrostatic effect.

## 2 Experimental Section

### 2.1 Synthesis of Materials

All reagents were of analytical grade and purchased from Sinopharm Chemical Reagent Co., Ltd., unless otherwise stated. Ba_0.5_Sr_0.5_Co_1–*y*_Fe_*y*_O_3–__δ_ (*y* = 0.2, 0.5, and 0.8) was prepared by the sol–gel method. Stoichiometric amounts of Ba(OH)_2_ and ethylenediaminetetraacetic acid (EDTA) were dispersed in water under stirring and heating, forming solution A; Sr(NO_3_)_2_, Fe(NO_3_)_3_·9H_2_O, and Co(NO_3_)_2_·6H_2_O were dispersed in water under stirring, forming solution B. Solution B and certain amounts of citric acid were successively added into solution A under stirring to obtain a mixed solution with the mole ratio of EDTA:citric acid:total metal ions = 1:1.5:1. NH_3_·H_2_O was used to adjust the pH of the solution to 6. The solution was transformed into a dry gel after evaporation. The dry gel was finally calcined at 950 °C for 5 h to prepare Ba_0.5_Sr_0.5_Co_1–*y*_Fe_*y*_O_3–__δ_ (*y* = 0.2, 0.5, and 0.8) powders. Ba_0.5_Sr_0.5_Co_0.8_Fe_0.2_O_3–__δ_, Ba_0.5_Sr_0.5_Co_0.5_Fe_0.5_O_3–__δ_, and Ba_0.5_Sr_0.5_Co_0.2_Fe_0.8_O_3–__δ_ were denoted as BSCF5582, BSCF5555, and BSCF5528, respectively. Cages like ZnO materials were successfully synthesized via a one-pot encapsulation–calcination method by using ZIF-8 (Zn) as a self-sacrificing template (Figures S1 and S2).

The GDC powders were synthesized by the citrate–nitrate combustion method as reported.^14^ GDC powder (0.6 g) was subjected to a uniaxial pressure of 185 MPa and sintered at 1500 °C for 10 h to obtain a GDC electrolyte disk with a diameter of 12.1 mm.

### 2.2 Sensor Fabrication and Characterization

For fabrication of the single SE sensor, a circular Pt paste (Sino-Platinum Co., Ltd., China) was coated on the GDC electrolyte disk and sintered at 1000 °C for 30 min, serving as the RE. ZnO, BSCF5582, BSCF5555, and BSCF5528 powders were mixed with organic binders (90 wt % α-terpineol and 10 wt % ethyl cellulose) in a mass ratio of 1:9 to prepare four SE pastes, respectively. Circular SE pastes were coated on the same side as the Pt RE of the GDC disk and then sintered at 600 °C (ZnO) or 950 °C (BSCF5582, BSCF5555, and BSCF5528) for 3 h, serving as the SE. Pt wires were connected to the SE and RE with some high-temperature silver paste (DAD-87, Shanghai Research Institute of Synthetic Resins, China) as the current collector. The single SE sensors based on ZnO, BSCF5582, BSCF5555, and BSCF5528 SE were denoted as the ZnO sensor, BSCF5582 sensor, BSCF5555 sensor, and BSCF5528 sensor, respectively. For fabrication of the dual SE sensor, circular ZnO SE pastes were coated on the GDC disk and sintered at 600 °C for 3 h, serving as SE1. Subsequently, other circular BSCF5582 SE pastes were coated on the same side of the disk and sintered at 950 °C for 3 h, serving as SE2. Pt wires were connected to the two SEs for collecting current. The dual SE sensor based on the ZnO SE and BSCF5582 SE was denoted as the ZnO-BSCF5582 sensor. Each electrode (SE or RE) had a diameter of 1.5 mm and was spaced 2.1 mm apart.

The phase composition and crystal structure of the sensing materials were determined by X-ray powder diffraction (XRD, Smartlab SE) using Cu Kα radiation. The morphology and microstructure of sensing materials were investigated by scanning electron microscopy (FE-SEM, Regulus8100) equipped with energy-dispersive X-ray spectrometry (EDX).

### 2.3 Sensor Test

The sensing performance measurements of sensors were conducted in a home-built sensor test system. The sensor was placed on a ceramic heating plate (Xinhe, Jiangsu) in the center of a quartz glass chamber with a volume of 2.72 L. The operating temperature of the sensor was controlled by regulating the voltage applied to the heating plate using a DC power supply (Wanptek, Shenzhen) and monitored by multiple thermocouples (ETA1080, Jiangsu) in real time. The open circuit voltage (OCV) between the SE and RE or dual SEs was measured by a Keysight electrometer (DAQ970A, U.S.A.) with the SE and RE/SE connecting to the positive and negative terminals of the electrometer, respectively. Appropriate amounts of certified analyte hydrogen balance with air (Nanjing Specialty Gas Co., Ltd.) were injected with a syringe for measurements of the potentiometric sensing performance. For recovery, the sensors were exposed to clean air again by removing the cover of the quartz glass chamber. The response value to hydrogen was defined as response = OCV (H_2_) – OCV (air), where OCV (H_2_) and OCV (air) were the OCV of the sensor in hydrogen and air, respectively. The sensitivity (*S*) was defined as the slope of linear fitting between the response and the hydrogen concentration logarithm in this work. The limit of detection (LOD) was determined as the lowest concentration at which the response is 3-fold higher than the root-mean-square noise (rms) of the baseline: LOD = 3 × rms/*S*.

## 3 Results and Discussion

### 3.1 Hydrogen Sensing Performance of BSCF5582

**Figure 1:**
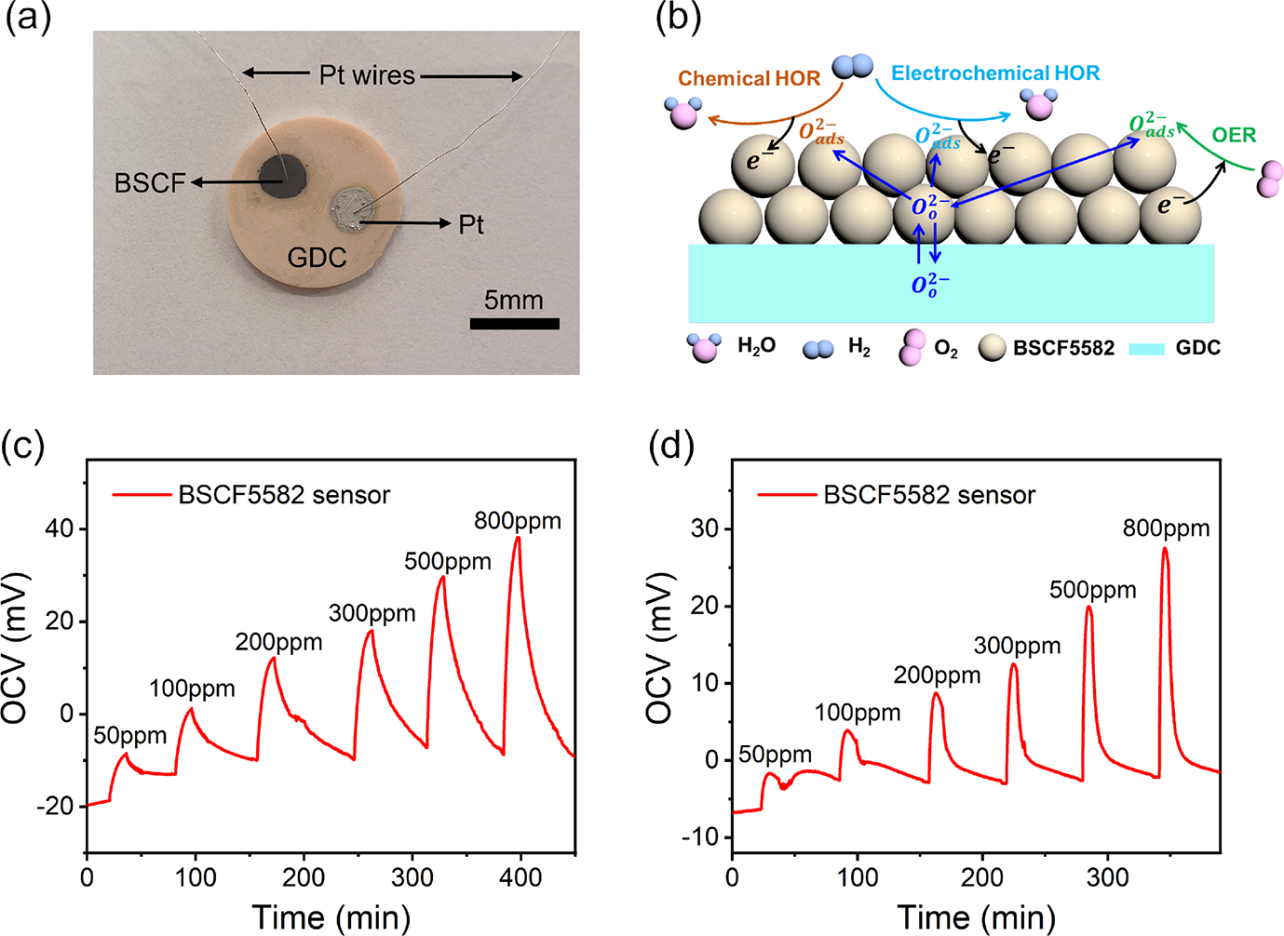
(a) Photograph and (b) schematic drawing showing the suggested sensing mechanism of the BSCF5582 sensor. Dynamic potentiometric response curves of the BSCF5582 sensor as a function of the hydrogen concentration in the range from 50 to 800 ppm at (c) 500 °C and (d) 550 °C.

To study the potentiometric hydrogen sensing performance of BSCF5582, a single SE sensor was fabricated (Figure 1a and see Figure S3 for characterization of the BSCF5582 SE material). As schematically depicted in Figure 1b, differing from pure electron-conducting semiconductors, the electrochemical hydrogen oxidation reaction (HOR) (H_2_ + O^2–^ = H_2_O + 2e^–^), the oxygen exchange reaction (OER) (O_2_ + 2e^–^ = 2O^2–^), and the chemical HOR (H_2_ + O_ads_^2–^ = H_2_O + 2e^–^) may take place simultaneously on the surface of mixed-ion-electron-conducting BSCF5582 electrode potential. Figure 1c and Figure 1d show the dynamic potentiometric response curves of the BSCF5582 sensor at 500 and 550 °C, respectively. Interestingly, the OCV shifted positively when the BSCF5582-based sensor was exposed to hydrogen-containing air, contrary to the negative OCV shifts of conventional semiconductor oxides.^6^ Such unusual responses have similarly been observed by other MIECs, such as SrFe_1–*x*_Ti_*x*_O_3–__δ_ and Sr_2_NiMoO_6–__δ_.^14^ The positive shifts increased with increasing hydrogen concentration and decreasing operating temperature. Upon removing the hydrogen, the OCV signals immediately moved toward the baseline and restored to the initial baseline after a long period. At a higher temperature of 550 °C, shorter response time and recovery time, i.e., larger response and recovery speeds, were observed (Figure S4). The higher temperature may have accelerated the equilibrium of relevant reactions as well as hydrogen adsorption and desorption on the surface of the BSCF electrode.^23^

**Figure 2:**
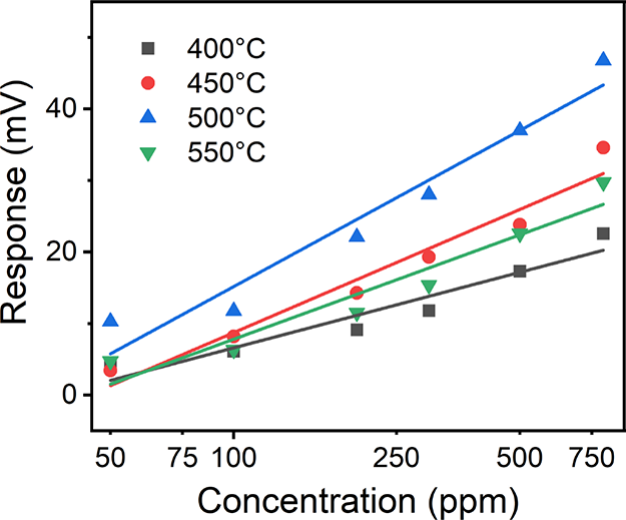
Response values of the BSCF5582 sensor as a function of the hydrogen concentration logarithm at 400–550 °C.

Figure 2 shows the potentiometric response of BSCF5582 as a function of the hydrogen concentration logarithm at 400–550 °C. As the temperature increased from 400 to 550 °C, the response values first increased and then decreased, demonstrating a parabolic change. As the operating temperature increased from 400 to 500 °C, the relevant electrochemical and chemical reactions of hydrogen on the BSCF surface could be enhanced, resulting in an increase in the response value (see the Sensing Mechanism section in Section 3.4 for details). When the operating temperature further increased to 550 °C, the desorption of hydrogen on the BSCF surface dominated and therefore the response value to hydrogen decreased again. The largest response was achieved at 500 °C, which was 37 mV for 500 ppm hydrogen. Linear relationships between response values and hydrogen concentration logarithms were observed. The sensitivity, i.e., the slope of the linear fitting, varied with operating temperature, which was consistent with the variation of response with temperature. The largest sensitivity of 31.2 mV/decade for 50–800 ppm hydrogen was obtained at 500 °C.

**Figure 3:**
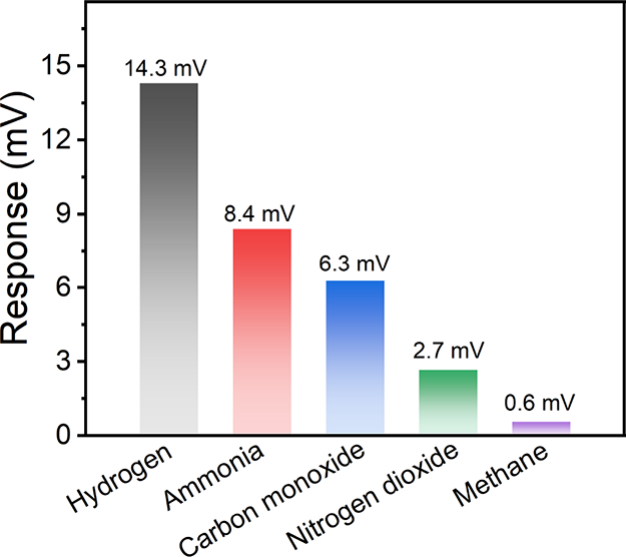
Cross-sensitivity of the BSCF5582 sensor to 100 ppm of various gases at 500 °C.

Figure 3 shows the cross-sensitivity of the BSCF5582 sensor to 100 ppm of various gases at 500 °C. Positive potentiometric response of the BSCF5582 sensor to ammonia, carbon monoxide, nitrogen dioxide, and methane was observed, similar to the response to hydrogen. The BSCF5582 sensor responded most sensitively to hydrogen, followed by ammonia and carbon monoxide, and negligibly to nitrogen dioxide and methane. The response to the most interfering gas, ammonia, was 8.4 mV, over 1.7 times smaller than that for hydrogen, suggesting good selective hydrogen sensing of the BSCF5582 sensor.

### 3.2 Enhanced Hydrogen Sensing Performance of ZnO-BSCF5582 Dual SEs

**Figure 4:**
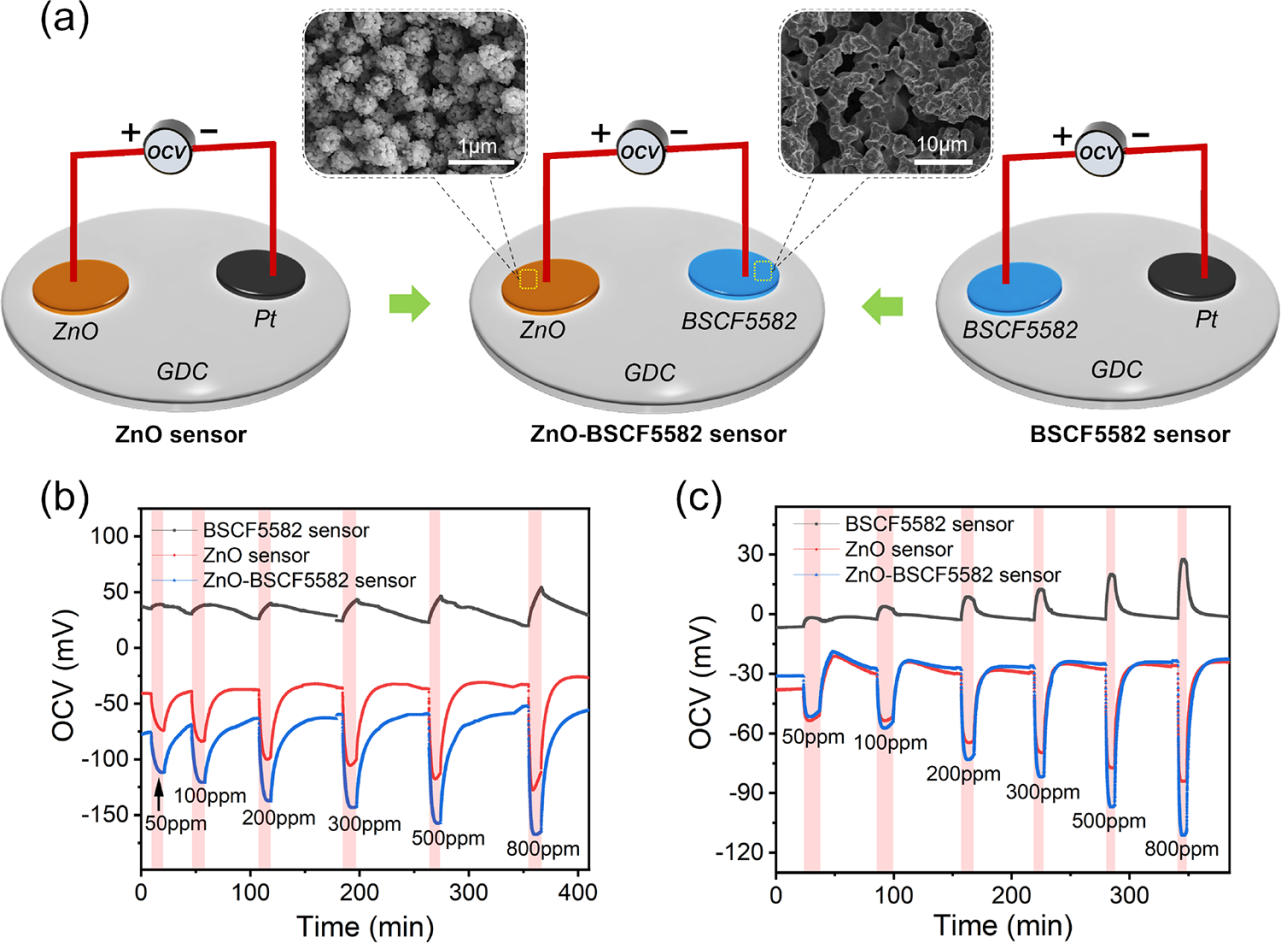
(a) Schematics of the ZnO sensor, BSCF5582 sensor, and ZnO-BSCF5582 sensor. (b, c) Dynamic potentiometric response curves of the three sensors as a function of the hydrogen concentration in the range from 50 to 800 ppm at (b) 450 °C and (c) 550 °C. Insets in panel (a) show SEM images of ZnO and BSCF5582 sensing materials.

The unusual potential polarity makes BSCF5582 advantageous for enhancing hydrogen sensing performance. To validate this idea, a dual SE sensor based on BSCF5582 and ZnO was constructed, while single SE sensors based on BSCF5582 (versus Pt) or ZnO (versus Pt) were also fabricated as comparisons (Figure 4a). Figure 4b,c shows dynamic potentiometric response curves of the two single SE sensors (ZnO sensor and BSCF5582 sensor) and a dual SE sensor (ZnO-BSCF5582 sensor) as a function of the hydrogen concentration in the range from 50 to 800 ppm at 450 and 550 °C. The negative shifts of OCV signals for ZnO were observed, which was similar to other studies and agreed well with non-Nernstian sensing behavior. The OCV signals of the ZnO-BSCF5582 sensor equal the difference of those for the ZnO sensor and BSCF5582 sensor, i.e., OCV (ZnO-BSCF5582) = OCV (ZnO) – OCV (BSCF5582). Owing to the opposite directions of OCV signal shifts for the ZnO sensor and BSCF5582 sensor, the shifted amounts of OCV signals for the ZnO-BSCF5582 sensor were significantly increased when compared to those for the single SE sensors. To highlight the advantages of BSCF5582 in improving hydrogen sensing, a similar sensor based on Co_3_O_4_ and ZnO dual SEs was also constructed (Figures S5 and S6). In comparison with the ZnO and Co_3_O_4_ single SE sensors, the shift amounts, response values, and sensitivity of the ZnO-Co_3_O_4_ sensor markedly decreased due to the same potential polarities of ZnO and Co_3_O_4_.

**Figure 5:**
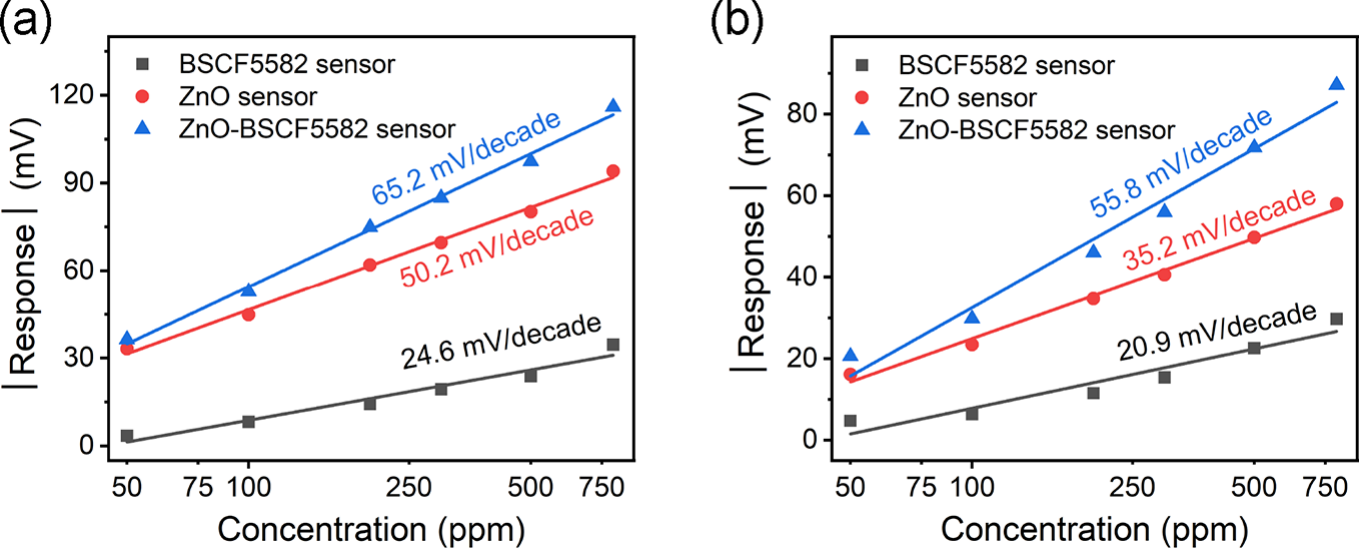
Response of the three sensors as a function of the hydrogen concentration in the range from 50 to 800 ppm at (a) 450 °C and (b) 550 °C.

Figure 5 shows the potentiometric response (absolute values are used for the sake of comparison) of the ZnO, BSCF5582, and ZnO-BSCF5582 sensors as a function of the hydrogen concentration logarithm at 450 and 550 °C. The response of the three sensors all varied linearly with the hydrogen concentration logarithm. The hydrogen response and sensitivity of the ZnO-BSCF5582 sensor were distinctly enhanced compared to those of the ZnO and BSCF5582 single SE sensors. A response of 97.3 mV was achieved at 450 °C for 500 ppm hydrogen, which were 1.2 and 4.1 times higher than those of a similar sensor with the ZnO SE and BSCF5582 SE, respectively. Similarly, the sensitivities for 50–800 ppm hydrogen were correspondingly 1.3 and 2.7 times larger at 450 °C. Owing to the enhanced sensitivity, the LOD for hydrogen can in principle be lowered. The calculated LOD of the ZnO-BSCF5582 sensor was 2.5 ppm, which were 3.1 and 6.5 times lower than those of the ZnO sensor and BSCF5582 sensor. At a higher temperature of 550 °C, a low LOD of 3.3 ppm was achieved, which were 4.8 and 3.9 times lower in contrast to those of the ZnO sensor and BSCF5582 sensor, respectively.

**Figure 6:**
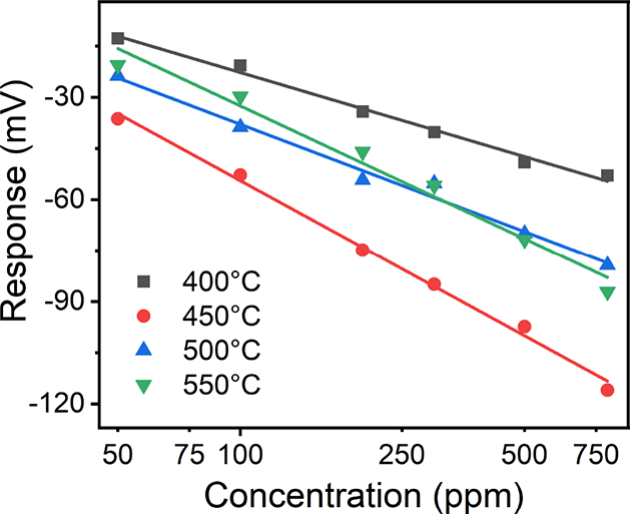
Response of the ZnO-BSCF5582 sensor as a function of the hydrogen concentration logarithm at 400–550 °C.

Figure 6 shows the response of the ZnO-BSCF5582 sensor as a function of the hydrogen concentration logarithm at 400–550 °C. It can be seen clearly that the response of the ZnO-BSCF5582 sensor first increased and then decreased as the operating temperature increased from 400 to 500 °C, while the response fluctuated only slightly when the temperature rose from 500 to 550 °C. The largest response was achieved at 450 °C, which were −36.3 and −84.9 mV for 50 and 300 ppm hydrogen, respectively. The sensitivities (the slope of linear fitting) at 400, 500, and 550 °C varied from 35.3 to 55.8 mV/decade. The largest sensitivity of −65.2 mV/decade for 50–800 ppm hydrogen was achieved at 450 °C.

Note that the response values of the dual SE sensor to hydrogen were obtained by superimposing the hydrogen sensing response of the two single sensing electrodes. For the ZnO-BSCF5582 sensor, the ZnO SE exhibited a higher response to hydrogen compared to the BSCF5582 SE and therefore dominated the total response (Figures 4 and 5). The optimal operating temperature of 450 °C for the ZnO-BSCF5582 sensor was dependent on the dominant ZnO SE rather than the BSCF5582 SE (BSCF5582 at 500 °C and ZnO at 450 °C). To maximize the hydrogen sensing performance of the dual SE sensor, two electrodes of opposite hydrogen sensing polarity with the same optimal operating temperatures should be selected to pair with each other in the future.

### 3.3 Effect of the Fe Doping Concentration on the Hydrogen Sensing Performance of BSCF

**Figure 7:**
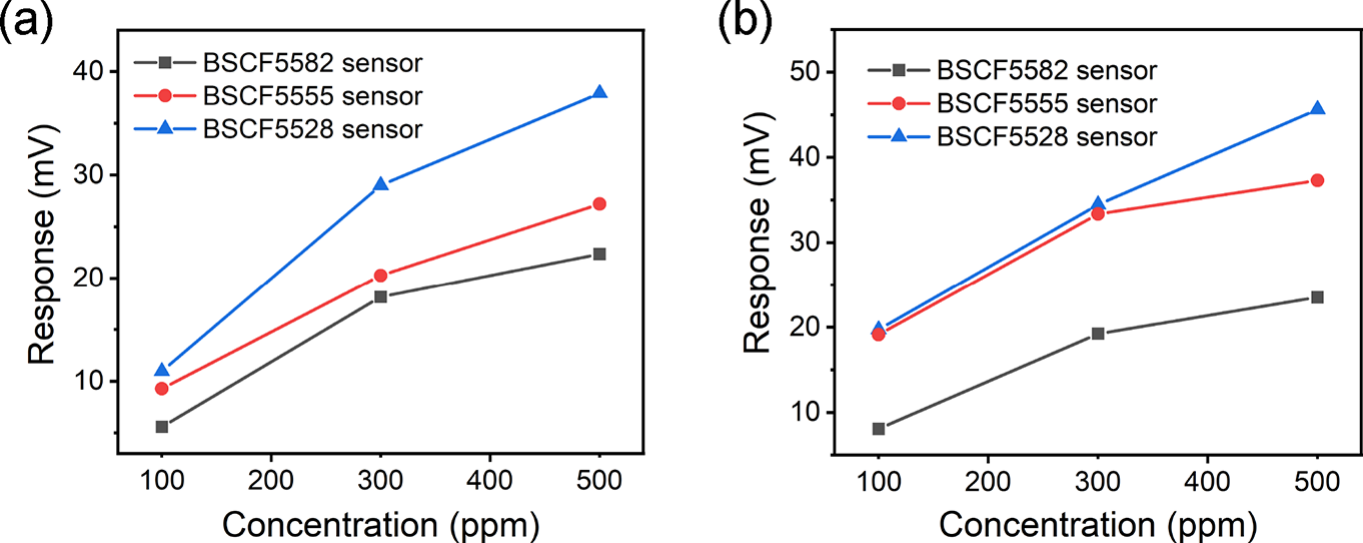
Response of the three BSCF sensors (BSCF5582, BSCF5555, and BSCF5528 sensors) as a function of the hydrogen concentration at (a) 350 °C and (b) 450 °C.

The thermal expansion coefficient and electrical conductivity were greatly affected by the B-site Fe doping level of BSCF.^21,24−30^ Therefore, modulation of the Fe doping concentration in the BSCF may further optimize the sensing performance for hydrogen and facilitate the development of highly sensitive dual SE hydrogen sensors. XRD patterns and SEM images of BSCF 5582, BSCF5555, and BSCF5528 in Figures S3 and S7 indicate that the three BSCFs were all perovskite oxides with single-phase cubic structures, and the variations in the amount of Fe elements had no impact on BSCF electrode morphology. The Fe/Co atomic ratios obtained by EDX were 0.26 for BSCF5582, 1.08 for BSCF5555, and 4.02 for BSCF5528, which were close to the recipes we designed in the synthesis section. Figure 7 compares the response of the three BSCF sensors (BSCF5582, BSCF5555, and BSCF5528 sensors) as a function of the hydrogen concentration at 350 and 450 °C. The potentiometric response values of BSCF to hydrogen increased monotonically with increasing Fe doping concentration, and the BSCF5528 sensor exhibited a higher response than the other two sensors. With the Fe/Co atomic ratio increased from 0.25 to 4, the responses to 500 ppm hydrogen raised by 69.6 and 94% at 350 and 450 °C, respectively. Therefore, among the three BSCF sensing materials, BSCF5528 in principle is the most desirable for the development of high-performance dual SE potentiometric sensors.

### 3.4 Sensing Mechanism

Mixed-ion-electron-conducting BSCF exhibited positive responses to hydrogen, which is opposite to that of electron-conducting semiconductors with negative responses such as ZnO. This unusual potential polarity of BSCF did not conform to conventional non-Nernstian sensing behaviors. The positive response to hydrogen can be explained as a result of a predominant surface electrostatic effect due to the dipoles and charged adsorbates of the BSCF.^31−33^ More specifically, in hydrogen-containing air, electrochemical HOR and OER occurred on the surface of BSCF, generating negative mixed potential (electrochemical, E_1_).^34,35^ In addition, chemical HOR takes place, resulting in the rise of the activity of electrons (c_e_) and the consumption of adsorbed oxygen.^36^ The former leads to a decrease in the electrode potential (electrical, E_2_) due to the upshift of the Fermi level, while the latter generates a positive electrostatic potential (electrostatic, E_3_).^31,37^ The E_3_ surpasses the sum of E_1_ and E_2_, thus yielding a positive response to hydrogen.

**Figure 8:**
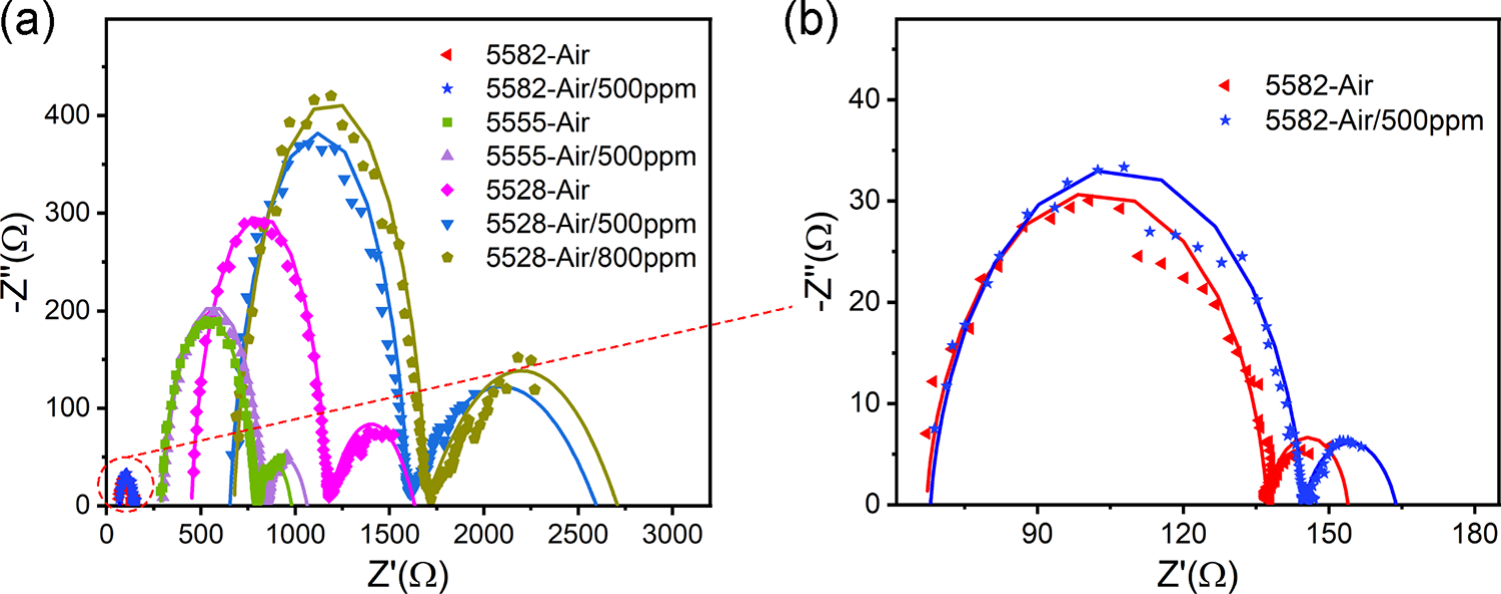
(a) Nyquist plots (symbols) and corresponding fits (solid lines) of the three BSCF sensors in different atmospheres at 550 °C. (b) Enlarged area in panel (a).

To further elucidate the sensing behaviors of BSCF with different Fe/Co atomic ratios, the impedance spectral measurements of three GDC-based BSCF symmetric electrodes in different atmospheres were conducted (Figure 8). The intercept of the high-frequency arc with the real axis corresponds to the ohmic resistance (*R*_o_) of the device, and that of the low frequency represents the total resistance (*R*_t_). The difference between *R*_t_ and *R*_o_ is the interface resistance (*R*_i_) of BSCF. As can be seen from Figure 8, upon exposure of the three devices to hydrogen-containing air, the *R*_o_ of p-type semiconducting BSCF all increased, indicating that the electron concentration due to the surface chemical reaction increased. On the other hand, the *R*_i_ of BSCF also increased significantly when hydrogen was introduced and further raised with increasing hydrogen concentration. It may imply that hydrogen hinders the OER on the BSCF. The variations of *R*_i_ for BSCF with the hydrogen concentration were similar to those of other MIECs reported.^15^ The resistance of BSCF increased dramatically with increasing Fe doping concentration, indicating a decrease in conductivity, which was consistent with previous studies.^22,28^ Moreover, when exposed to a hydrogen-containing atmosphere, the magnitudes of *R*_o_ and *R*_i_ variations increased with increasing Fe doping concentration. This suggests that an increase in Fe doping could make a more significant rise in electronic activity and a more pronounced inhibition of the OER by hydrogen. As a result, E_1_, E_2,_ and E_3_ should all increase, and E_3_ increases more than E_1_ and E_2_ increases, leading to an enhancement in the positive hydrogen response.

The above results show that the RE was of crucial importance for the performance of mixed potential hydrogen sensors. In comparison with conventional electronic conducting semiconductor oxides such as Co_3_O_4_, BSCF with unusual potential polarity was desirable as a substitute for the Pt RE for improving the hydrogen sensing performance, including enhanced response and sensitivity and reduced LOD, beneficial to achieving highly sensitive hydrogen detection. Moreover, the hydrogen sensing response of BSCF can be raised by increasing the B-site Fe/Co atomic ratio, which provides an opportunity to further optimize the hydrogen sensing performance.

## 4 Conclusions

A low-cost planar dual SE hydrogen sensor was fabricated by substitution of the Pt RE with another semiconductor oxide SE. The hydrogen sensing response was partially counteracted and markedly reduced when paired with conventional electronic conducting SEs of the same potential polarity such as ZnO and Co_3_O_4_. The mixed conducting Ba_0.5_Sr_0.5_Co_0.8_Fe_0.2_O_3–__δ_ exhibited an unusual potential polarity to hydrogen. Enhanced hydrogen sensing including a higher response, larger sensitivity, and lower LOD was achieved by pairing unusual Ba_0.5_Sr_0.5_Co_0.8_Fe_0.2_O_3–__δ_ with ZnO of opposite potential polarity. A high response of 97.3 mV for 500 ppm hydrogen and a low LOD of 2.5 ppm were obtained by the dual SE sensor at 450 °C. The potentiometric response values to hydrogen increased monotonically with increasing Fe doping concentration in Ba_0.5_Sr_0.5_Co_1–*y*_Fe_*y*_O_3–__δ_ (*y* = 0.2, 0.5, and 0.8). With the Fe/Co atomic ratio increased from 0.25 to 4, the responses to 500 ppm hydrogen raised by 69.6 and 94% at 350 and 450 °C, respectively. The sensing behaviors of unusual Ba_0.5_Sr_0.5_Co_1–*y*_Fe_*y*_O_3–__δ_ may be ascribed to the predominant surface electrostatic effect.

## Supporting Information Available

(Figure S1) Schematic diagram of the synthesis procedure for cage-like ZnO materials; (Figure S2) XRD pattern of cage-like ZnO sensing material; (Figure S3) XRD pattern of the BSCF5582 sensing material; (Figure S4) variations of response time and recovery time for the BSCF5582 sensor with the hydrogen concentration at 500 and 550 °C; (Figure S5) XRD pattern and SEM image of Co_3_O_4_ sensing material; (Figure S6) dynamic potentiometric response curves and response values of the three sensors as a function of the hydrogen concentration from 50 to 800 ppm at 400 °C; (Figure S7) XRD patterns of BSCF5555 and BSCF5528 sensing materials; SEM images of BSCF5555 and BSCF5528 sensing materials (PDF)

## References

[ref1] Kopasz J. P. Fuel cells and odorants for hydrogen. Int. J. Hydrogen Energy 2007, 32 (13), 2527–2531. 10.1016/j.ijhydene.2006.11.001.

[ref2] Wischmeyer T.; Stetter J. R.; Buttner W. J.; Patel V.; Peaslee D. Characterization of a selective, zero power sensor for distributed sensing of hydrogen in energy applications. Int. J. Hydrogen Energy 2021, 46 (61), 31489–31500. 10.1016/j.ijhydene.2021.07.015.

[ref3] Zhang X.; Zhang X.; Li X.; Liu Q.; Zhang Y.; Liang Y.; Liu Y.; Peng W. The nanophotonic machinal cavity and its hydrogen sensing application. Sens. Actuator B-Chem. 2022, 367, 132095 10.1016/j.snb.2022.132095.

[ref4] Ramaiyan K.; Tsui L. k.; Brosha E. L.; Kreller C.; Stetter J. R.; Russ T.; Du W.; Peaslee D.; Hunter G.; Xu J.; Makel D.; Garzon F.; Mukundan R. Recent Developments in Sensor Technologies for Enabling the Hydrogen Economy. ECS Sens. Plus 2023, 2, 045601 10.1149/2754-2726/ad0736.

[ref5] Gorbova E.; Balkourani G.; Molochas C.; Sidiropoulos D.; Brouzgou A.; Demin A.; Tsiakaras P. Brief Review on High-Temperature Electrochemical Hydrogen Sensors. Catalysts 2022, 12 (12), 1647 10.3390/catal12121647.

[ref6] Miura N.; Sato T.; Anggraini S. A.; Ikeda H.; Zhuiykov S. A review of mixed-potential type zirconia-based gas sensors. Ionics 2014, 20 (7), 901–925. 10.1007/s11581-014-1140-1.

[ref7] Liu F.; Wang B.; Yang X.; Liang X.; Sun P.; Chuai X.; Gao Y.; Liu F.; Lu G. Sub-ppm YSZ-based mixed potential type acetone sensor utilizing columbite type composite oxide sensing electrode. Sens. Actuator B-Chem. 2017, 238, 928–937. 10.1016/j.snb.2016.06.171.

[ref8] Dai L.; Liu Y.; Meng W.; Yang G.; Zhou H.; He Z.; Li Y.; Wang L. Ammonia sensing characteristics of La_10_Si_5_MgO_26_-based sensors using In_2_O_3_ sensing electrode with different morphologies and CuO reference electrode. Sens. Actuator B-Chem. 2016, 228, 716–724. 10.1016/j.snb.2016.01.106.

[ref9] Ritter T.; Lattus J.; Hagen G.; Moos R. Effect of the Heterogeneous Catalytic Activity of Electrodes for Mixed Potential Sensors. J. Electrochem. Soc. 2018, 165 (16), B795–B803. 10.1149/2.0181816jes.

[ref10] Jin H.; Plashnitsa V. V.; Breedon M.; Miura N. Compact YSZ-Rod-Based Hydrocarbon Sensor Utilizing Metal-Oxide Sensing-Electrode and Mn-Based Reference-Electrode Combination. Electrochem. Solid St. 2011, 14 (6), J23–J25. 10.1149/1.3568883.

[ref11] Lee I.; Jung B.; Park J.; Lee C.; Hwang J.; Park C. O. Mixed potential NH_3_ sensor with LaCoO_3_ reference electrode. Sens. Actuator B-Chem. 2013, 176, 966–970. 10.1016/j.snb.2012.09.009.

[ref12] Yang L.; Wu C.; Zhang Y.; Xiao B.; Jiao A.; Li K.; Chen T.; Zhan R.; Lin H. Enhancement of Ammonia Sensors Using In_2_O_3_ Sensing Electrode by Adjusting Particle Size and NiCo_2_O_4_ Reference Electrode. J. Electrochem. Soc. 2022, 169 (8), 087505 10.1149/1945-7111/ac83f4.

[ref13] Hao X.; Li W.; Lu Q.; Wang T.; Wang B.; Liu T.; Liang X.; Liu F.; Wang C.; Lu G. Specificity improvement of the YSZ-based mixed potential gas sensor for acetone and hydrogen sulfide detection. Sens. Actuator B-Chem. 2021, 341, 129292 10.1016/j.snb.2020.129292.

[ref14] Zhang Y.; Liu Y.; Wang L.; Zhou H.; Meng W.; Li Y.; He Z.; Dai L. A mixed-potential type NH_3_ sensors based on spinel Zn_2_SnO_4_ sensing electrode. Sens. Actuator B-Chem. 2022, 367, 132154 10.1016/j.snb.2022.132154.

[ref15] Zhang Z.; Yi J.; Han H.; Meng Y.; Zhang H.; Jiang Y. Electrochemical Response of Mixed Conducting Perovskite Enables Low-Cost High-Efficiency Hydrogen Sensing. ACS Appl. Mater. Interfaces 2022, 14 (29), 33580–33588. 10.1021/acsami.2c09642. 35849478

[ref16] Zhang Y.; Xiao B.; Yang L.; Jiao A.; Li K.; Wu C.; Zhan R.; Huang Z.; Lin H. Sensitivity and selectivity enhancement of the YSZ-based mixed-potential ammonia sensors with flame-spray-made double-sensing electrodes. Sens. Actuator B-Chem. 2021, 344, 130165 10.1016/j.snb.2021.130165.

[ref17] Sun S.; Cheng Z. Effects of H_2_O and CO_2_ on Electrochemical Behaviors of BSCF Cathode for Proton Conducting IT-SOFC. J. Electrochem. Soc. 2017, 164 (2), F81–F88. 10.1149/2.0611702jes.

[ref18] Han D.; Tan X.; Yan Z.; Li Q.; Liu S. New morphological Ba_0.5_Sr_0.5_Co_0.8_Fe_0.2_O_3-α_ hollow fibre membranes with high oxygen permeation fluxes. Ceram. Int. 2013, 39 (1), 431–437. 10.1016/j.ceramint.2012.06.044.

[ref19] Risch M.; Stoerzinger K. A.; Maruyama S.; Hong W. T.; Takeuchi I.; Shao-Horn Y. La_0.8_Sr_0.2_MnO_3-δ_ Decorated with Ba_0.5_Sr_0.5_Co_0.3_Fe_0.2_O_3-δ_: A Bifunctional Surface for Oxygen Electrocatalysis with Enhanced Stability and Activity. J. Am. Chem. Soc. 2014, 136 (14), 5229–5232. 10.1021/ja5009954. 24649849

[ref20] Zhang H.; Zhang Z.; Li Z.; Han H.; Song W.; Yi J. A chemiresistive-potentiometric multivariate sensor for discriminative gas detection. Nat. Commun. 2023, 14 (1), 3495–3495. 10.1038/s41467-023-39213-x. 37311822 PMC10264437

[ref21] Chen Z.; Ran R.; Zhou W.; Shao Z.; Liu S. Assessment of Ba_0.5_Sr_0.5_Co_1-y_Fe_y_O_3-δ_ (y = 0.0–1.0) for prospective application as cathode for IT-SOFCs or oxygen permeating membrane. Electrochim. Acta 2007, 52 (25), 7343–7351. 10.1016/j.electacta.2007.06.010.

[ref22] Zhao H.; Shen W.; Zhu Z.; Li X.; Wang Z. Preparation and properties of Ba_x_Sr_1-x_Co_y_Fe_1-y_O_3-δ_ cathode material for intermediate temperature solid oxide fuel cells. J. Power Sources 2008, 182 (2), 503–509. 10.1016/j.jpowsour.2008.04.046.

[ref23] Liu F.; Wang J.; Li B.; You R.; Wang C.; Jiang L.; Yang Y.; Yan X.; Sun P.; Lu G. Ni-based tantalate sensing electrode for fast and low detection limit of acetone sensor combining stabilized zirconia. Sens. Actuator B-Chem. 2020, 304, 127375 10.1016/j.snb.2019.127375.

[ref24] Lim Y. H.; Lee J.; Yoon J. S.; Kim C. E.; Hwang H. J. Electrochemical performance of Ba_0.5_Sr_0.5_Co_x_Fe_1-x_O_3-δ_ (x = 0.2–0.8) cathode on a ScSZ electrolyte for intermediate temperature SOFCs. J. Power Sources 2007, 171 (1), 79–85. 10.1016/j.jpowsour.2007.05.050.

[ref25] Jung J.-I.; Edwards D. D. X-ray photoelectron study on Ba_0.5_Sr_0.5_Co_x_Fe_1-x_O_3-δ_ (BSCF: x = 0.2 and 0.8) ceramics annealed at different temperature and pO_2_. J. mater. Sci. 2011, 46 (23), 7415–7422. 10.1007/s10853-011-5704-4.

[ref26] Jung J.-I.; Misture S. T.; Edwards D. D. The electronic conductivity of Ba_0.5_Sr_0.5_Co_x_Fe_1-x_O_3-δ_ (BSCF: x = 0 similar to 1.0) under different oxygen partial pressures. J. Electroceram. 2010, 24 (4), 261–269. 10.1007/s10832-009-9567-x.

[ref27] Kotomin E. A.; Mastrikov Y. A.; Kuklja M. M.; Merkle R.; Roytburd A.; Maier J. First principles calculations of oxygen vacancy formation and migration in mixed conducting Ba_0.5_Sr_0.5_Co_1-y_Fe_y_O_3-δ_ perovskites. Solid State Ionics 2011, 188 (1), 1–5. 10.1016/j.ssi.2010.10.011.

[ref28] Yáng Z.; Harvey A. S.; Infortuna A.; Schoonman J.; Gauckler L. J. Electrical conductivity and defect chemistry of Ba_x_Sr_1-x_Co_y_Fe_1-y_O_3-δ_ perovskites. J. Solid State Electr. 2011, 15 (2), 277–284. 10.1007/s10008-010-1208-4.

[ref29] Mahadik P. S.; Shirsat A. N.; Saha B.; Sitapure N.; Tyagi D.; Varma S.; Wani B. N.; Bharadwaj S. R. Chemical compatibility study of BSCF cathode materials with proton-conducting BCY/BCZY/BZY electrolytes. J. Therm. Anal. Calorim. 2019, 137 (6), 1857–1866. 10.1007/s10973-019-08082-2.

[ref30] Lannelongue P.; Le Vot S.; Fontaine O.; Sougrati M.-T.; Crosnier O.; Brousse T.; Favier F. Investigation of Ba_0.5_Sr_0.5_Co_x_Fe_1-x_O_3-δ_ as a pseudocapacitive electrode material with high volumetric capacitance. Electrochim. Acta 2018, 271, 677–684. 10.1016/j.electacta.2018.03.173.

[ref31] Feng Z. A.; Balaji Gopal C.; Ye X.; Guan Z.; Jeong B.; Crumlin E.; Chueh W. C. Origin of Overpotential-Dependent Surface Dipole at CeO_2-x_/Gas Interface During Electrochemical Oxygen Insertion Reactions. Chem. Mater. 2016, 28 (17), 6233–6242. 10.1021/acs.chemmater.6b02427.

[ref32] De Souza R. A. Limits to the rate of oxygen transport in mixed-conducting oxides. J. Mater. Chem. A 2017, 5 (38), 20334–20350. 10.1039/C7TA04266C.

[ref33] Feng Z. A.; El Gabaly F.; Ye X.; Shen Z.-X.; Chueh W. C. Fast vacancy-mediated oxygen ion incorporation across the ceria-gas electrochemical interface. Nat. Commun. 2014, 5, 4374 10.1038/ncomms5374. 25007038

[ref34] Yi J.; Han H. Analysis of factors affecting response for mixed potential gas sensors. Electrochim. Acta 2021, 379, 138129 10.1016/j.electacta.2021.138129.

[ref35] Zhang H.; Yi J.; Zhang Z.; Zhang H. The relation between mixed-potential hydrogen response and electrochemical activities for perovskite oxides. Sens. Actuator B-Chem. 2022, 352, 130988 10.1016/j.snb.2021.130988.

[ref36] Koo W.-T.; Cho H.-J.; Kim D.-H.; Kim Y. H.; Shin H.; Penner R. M.; Kim I.-D. Chemiresistive Hydrogen Sensors: Fundamentals, Recent Advances, and Challenges. ACS Nano 2020, 14 (11), 14284–14322. 10.1021/acsnano.0c05307. 33124428

[ref37] Fleig J.; Merkle R.; Maier J. The p(O_2_) dependence of oxygen surface coverage and exchange current density of mixed conducting oxide electrodes: model considerations. Phys. Chem. Chem. Phys. 2007, 9 (21), 2713–2723. 10.1039/b618765j. 17627315

