# Supplementary material for: Enhanced Potentiometric Hydrogen Sensing Response Based on the Ba0.5Sr0.5Co1–yFeyO3–δ Electrode with Unusual Polarity
Source: ACS Omega. 2024 Feb 14;9(8):8885–92. doi: 10.1021/acsomega.3c06833 (PMC10905735; doi:10.1021/acsomega.3c06833)
Supplement: Supplementary file 1 — ao3c06833_si_001.pdf [file ao3c06833_si_001.pdf]

# Supporting Information

# Enhanced potentiometric hydrogen sensing response based on $Ba_{0.5}Sr_{0.5}Co_{1-y}Fe_yO_{3-\delta}$ electrode with unusual polarity

Hong Zhang[1], Yanqing Liu[1], Hailin Su[1], Yuelong Zhu[1], Haowei Zhu[1], Shibin Nie[2,*], Liangji Xu[3]

[1]*Joint National-Local Engineering Research Centre for Safe and Precise Coal Mining, Anhui University of Science and Technology, Huainan, Anhui, 232001, P.R. China*

[2]*College of Public Safety and Emergency Management, Anhui University of Science and Technology, Hefei, Anhui, 231131, PR China*

[3]*Institute of Energy, Hefei Comprehensive National Science Center, Hefei, Anhui 230051, PR China*

[*] *Corresponding Author. E-mail address: nieshibin88@163.com*

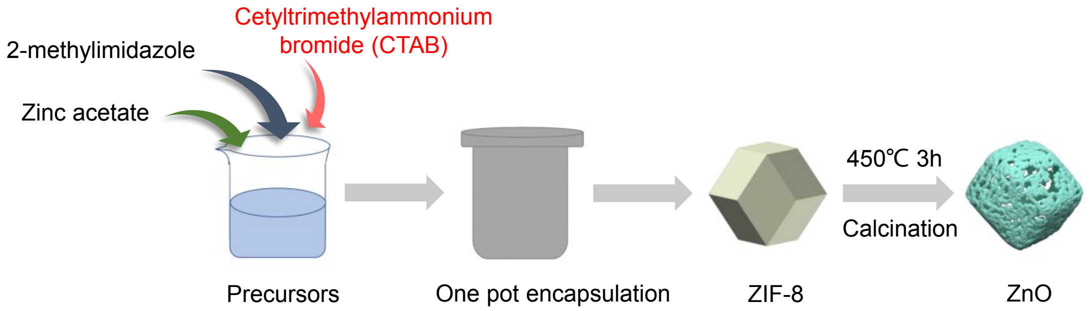


**Figure S1.** Schematic diagram of synthesis procedure for cages like ZnO materials.

ZnO cages produced from ZIF-8 were created using a one-pot encapsulation-calcination technique, as shown in Figure S1. Firstly, 2 mmol (366.96 mg) of zinc acetate and 60 mmol (4.93 mg) of 2-methylimidazole were sequentially dissolved in 3.6 mol (64.5 ml) of deionized water under stirring. Secondly, CTAB of 0.35 wt% (246.89 mg) was added to prepare the precursors for one pot encapsulation. The precursors were subsequently placed in a 60 mL Teflon-lined stainless steel autoclave and heated for 24 h at 120°C. Centrifugation was used to separate the precipitates, which were then carefully cleaned with deionized water three times and dried for 24 h at 60°C. At last, by calcining the precipitates at 450°C for three hours at a heating rate of 1 °C/min, cage-like ZnO structures were prepared.

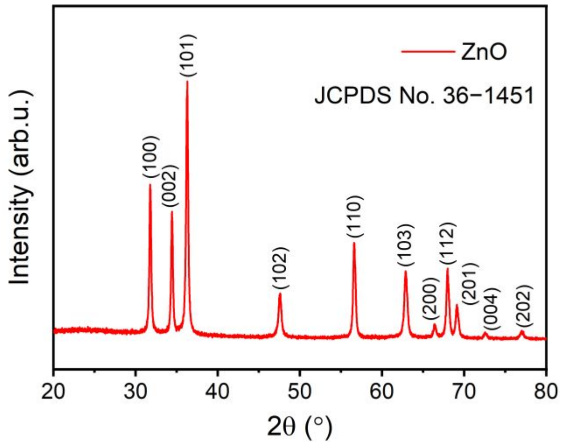


**Figure S2.** XRD pattern of cage-like ZnO sensing material.

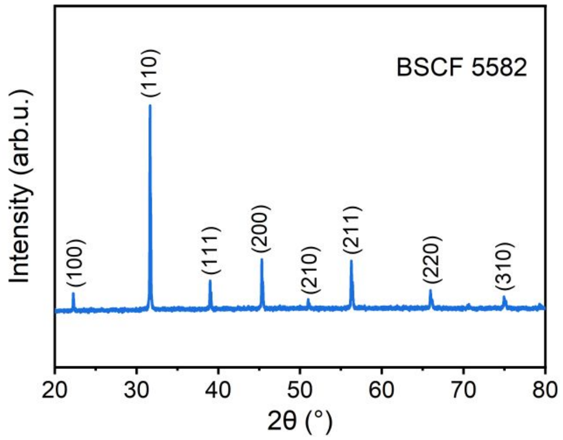


**Figure S3.** XRD pattern of the BSCF5582 sensing material.

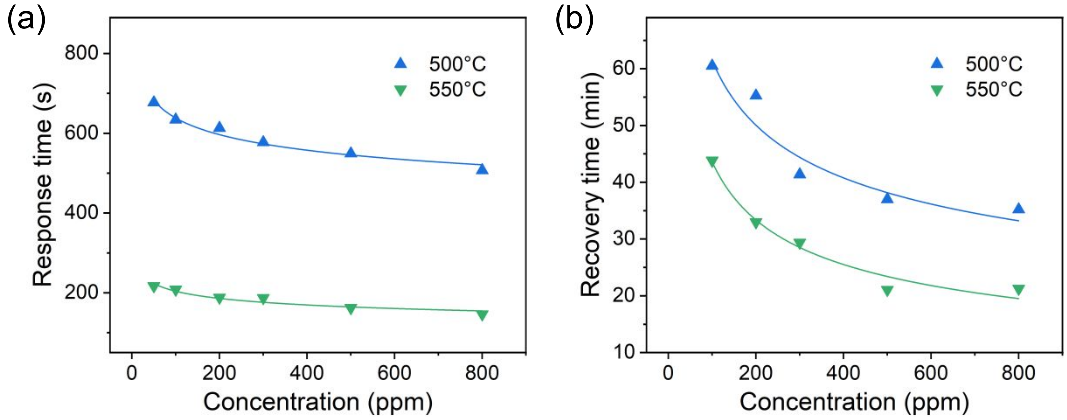


**Figure S4.** Variations of (a) response time and (b) recovery time for the BSCF5582 sensor with hydrogen concentration at 500°C and 550°C.

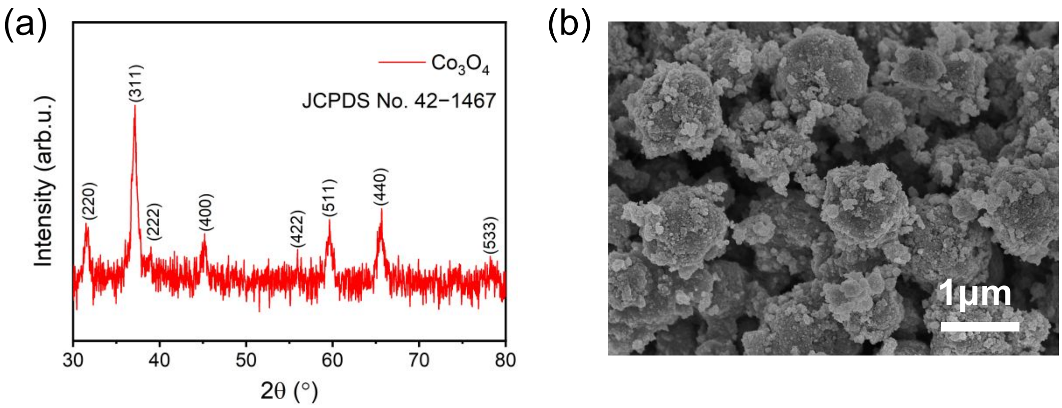


**Figure S5.** (a) XRD pattern and (b) SEM image of $Co_3O_4$ sensing material.

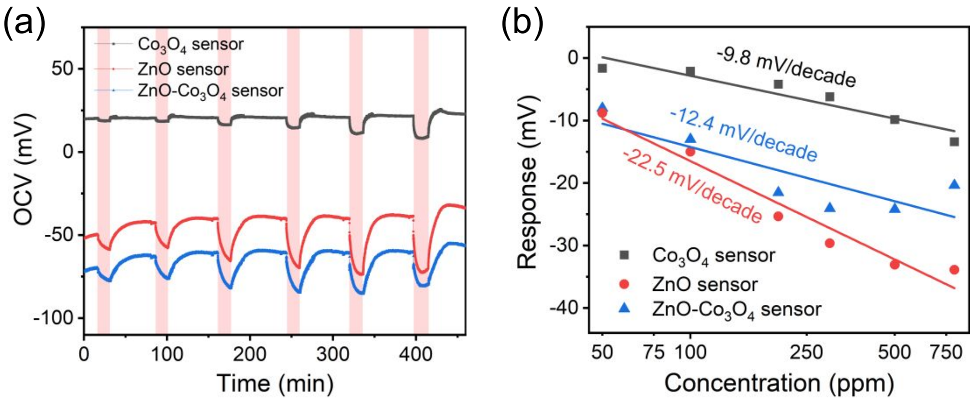


**Figure S6** (a) Dynamic potentiometric response curves and (b) response values of the three sensors as a function of hydrogen concentration from 50 to 800 ppm at 400°C.

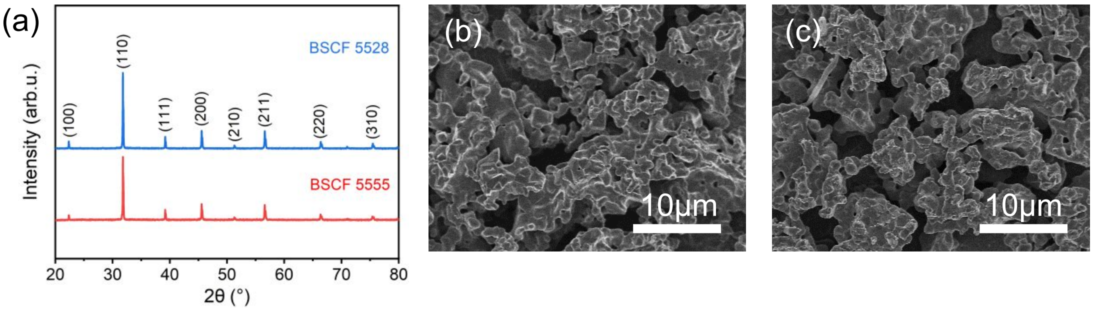


**Figure S7** (a) XRD patterns of BSCF5555 and BSCF5528 sensing materials. (b-c) SEM images of (b) BSCF5555 and (c) BSCF5528 sensing materials.
